# The Role of Blood-Derived Factors in Protection and Regeneration of Aged Tissues

**DOI:** 10.3390/ijms23179626

**Published:** 2022-08-25

**Authors:** Anna L. Höving, Kazuko E. Schmidt, Barbara Kaltschmidt, Christian Kaltschmidt, Cornelius Knabbe

**Affiliations:** 1 Heart and Diabetes Centre NRW, Institute for Laboratory and Transfusion Medicine, Ruhr-University Bochum, 32545 Bad Oeynhausen, Germany; 2 Department of Cell Biology, Faculty of Biology, Bielefeld University, 33615 Bielefeld, Germany; 3 AG Molecular Neurobiology, Faculty of Biology, Bielefeld University, 33615 Bielefeld, Germany

**Keywords:** adult cardiac stem cells, young blood, stem cell viability, blood serum, tissue regeneration, tissue protection

## Abstract

Tissue regeneration substantially relies on the functionality of tissue-resident endogenous adult stem cell populations. However, during aging, a progressive decline in organ function and regenerative capacities impedes endogenous repair processes. Especially the adult human heart is considered as an organ with generally low regenerative capacities. Interestingly, beneficial effects of systemic factors carried by young blood have been described in diverse organs including the heart, brain and skeletal muscle of the murine system. Thus, the interest in young blood or blood components as potential therapeutic agents to target age-associated malignancies led to a wide range of preclinical and clinical research. However, the translation of promising results from the murine to the human system remains difficult. Likewise, the establishment of adequate cellular models could help to study the effects of human blood plasma on the regeneration of human tissues and particularly the heart. Facing this challenge, this review describes the current knowledge of blood plasma-mediated protection and regeneration of aging tissues. The current status of preclinical and clinical research examining blood borne factors that act in stem cell-based tissue maintenance and regeneration is summarized. Further, examples of cellular model systems for a more detailed examination of selected regulatory pathways are presented.

## 1. Introduction

**Figure 1:**
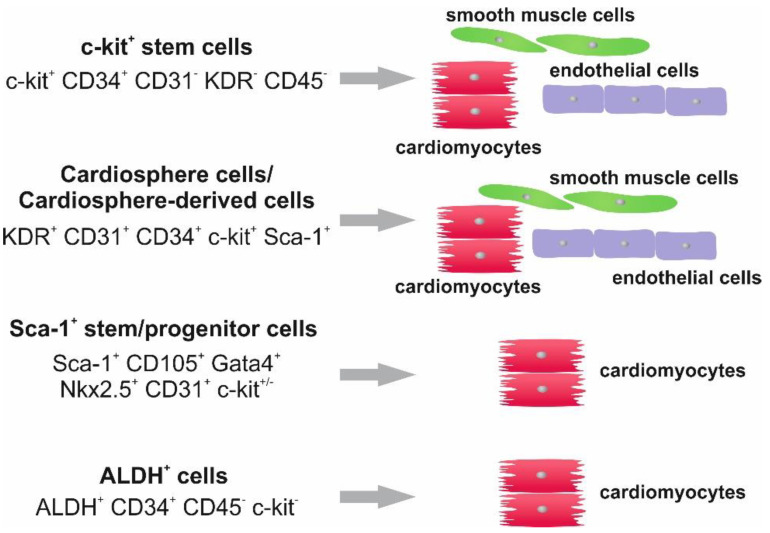
Overview about previously isolated and expanded cardiac stem and progenitor populations from the adult human heart. Marker profiles as well as differentiation capacities are depicted. See Refs. [14,22,23,24,25,26,29,30].

The aging process is associated with a progressive decline in tissue functionality, leading to severe age-associated degenerative diseases. The maintenance of tissue functionality as well as the regeneration of injured tissue is ensured by the activity of endogenous stem cell populations [1,2,3,4]. However, it has been shown that tissue aging is connected with diminished activity of tissue-resident progenitor cells or stem cells [5,6,7]. Cellular senescence occurs due to telomere-shortening, mitochondrial dysfunction or oxidative stress, inducing DNA-damage and the subsequent expression of cell cycle inhibitors [8]. In detail, this process and the resulting cellular responses are dependent on cell type, age or the cellular microenvironment. For instance, Anderson and colleagues described an age-dependent senescence mechanism in mouse cardiomyocytes that consists of telomere damage but not telomere shortening [9]. In mouse models, the senolytic drug ABT263 (Navitoclax) was able to reduce this age-associated telomere dysfunction in cardiomyocytes by inhibiting proteins of the Bcl-2 family of anti-apoptotic factors [10]. An age-independent senescence mechanism is induced by diabetes mellitus type 2, where an increased production of reactive oxygen species (ROS) leads to an increased number of senescent cardiac stem or progenitor cells in human patients accompanied by reduced proliferation and limited stem cell functions such as sphere-forming capacities and differentiation in the myogenic lineage [11]. In a mouse model, this diabetes type 2-induced senescent phenotype could be successfully resorted by the application of the senolytics dasatinib and quercetin, which likewise induce apoptosis of senescent cells [11]. Currently, a clinical trial is planned to investigate a beneficial effect of the senolytic drug Fisetin as an addition to the treatment with bone marrow stem cells for osteoarthritis patients [12]. Facing cardiac regeneration, the existence of endogenous stem cell populations in the adult murine heart was first reported in 2003 [13], and multipotent human cardiac stem cells were described in 2007 [14]. A range of studies followed, describing other cardiac stem and progenitor cell populations based on the expression of different stem cell markers [15,16,17,18,19,20,21,22,23,24,25,26]. Excellent overviews about mammalian cardiac stem cell populations were published in 2018 and 2019 [27,28]. In addition, Figure 1 focuses on the currently known stem and progenitor cell populations that can be identified in the adult human heart. In detail, adult cardiac stem or progenitor cells can be isolated from human heart tissue based on their capacity to grow on the single cell level or the expression of the cell surface marker Sca-1 [22,29]. These cells were further described by Smits and colleagues to express the mesenchymal stem cell marker CD105, the endothelial cell marker CD31, as well as the stem cell marker Sca-1. The stem cell marker c-Kit (CD117) was detectable only in a minor fraction of cells. On a functional level, these cardiac progenitor cells successfully differentiated into spontaneously beating cardiomyocytes [22]. In 2012, Chimenti and colleagues provided a detailed protocol for the isolation and expansion of so-called cardiosphere-derived cells (CDCs), a human cardiac stem and progenitor population expressing c-kit with the potential to give rise to cells from the cardiogenic and vasculogenic lineages [30]. Cardiospheres (CSs) and CDCs were further described by Barile and coworkers with an extended marker profile showing markers from the mesenchymal lineage as well as markers for perivascular cells [23]. Another strategy for the isolation of human cardiac stem cells is based on the activity of aldehyde dehydrogenase (ALDH). ALDH+ cells express a wide range of pluripotency-associated genes, such as Oct-4, Nanog, c-Myc, Klf-4 and lin-28, likewise showing a robust capacity to differentiate into cardiomyocytes [24] (Figure 1).

The composition of marker proteins indicating bona fide cardiac stem cells has been widely discussed. While the first reports about the identification of adult cardiac stem cells focused on c-kit-positive (c-kit^+^) cells [14], other reports challenged the existence of adult cardiac stem cells in general, claiming that c-kit^+^ cells do not induce cardiac repair in a sufficient manner. For example, lineage tracing using c-kit^+^/Cre mice revealed that endogenous c-kit^+^ cells indeed give rise to new cardiomyocytes, however, at a low level of approximately 0.003 percent [31]. This experimental approach was challed into question later, showing that the used c-kit^+^/Cre system does not reliably track a Lin^−^c-kit^low^ cardiac stem cell population [32]. Moreover, it has been shown that the knock-in of the Cre recombinase to the c-kit locus generates c-kit haploinsufficient mice with an impaired endogenous cardiac repair after injury compared to c-kit diploid mice [33]. However, more detailed experiments showed that additional depletion of CD45^+^ cells resulted in a c-kit^+^CD45^−^ subpopulation that is capable of supporting functional myocardial regeneration in a mouse model of myocardial injury [34]. However, despite the existence of endogenous adult cardiac stem cell populations, the regenerative capacities of the aging human heart are limited [35]. Interestingly, proliferating cardiomyocytes can be detected in the fetal heart [36], in contrast to a generally low proliferation rate in the adult heart with a yearly turnover of 0.5 to 1% [37,38]. Thus, the in vivo contribution of resident stem and progenitor cell populations to cardiac repair is unclear and much discussed [39,40]. Notably, attempts to apply other non-cardiac stem cell populations were only partially successful, and the mode of action of these transplanted stem cell populations in heart regeneration remained elusive. For instance, contradictory results were reported regarding the amount of repaired myocardium with differentiating transplanted cells [41,42]. The intracoronary application of autologous bone marrow-derived mononuclear cells was first recognized as a highly promising strategy, but the initial clinical results were difficult to reproduce [43,44]. In addition, the inflammatory microenvironment at the infarct site was considered to inhibit successful stem cell engraftment [45]. Currently, the majority of actively recruiting clinical trials using adult stem or progenitor cells for myocardial repair focus on allogenic umbilical cord-derived stem cells [46,47,48,49]. Later, the field of research for stem cell-based tissue replacements moved from autologous adult stem cells to pluripotent embryonic or reprogrammed stem cells, which need to be differentiated in vitro to avoid teratoma formation. Using modern tissue engineering techniques, induced pluripotent stem cell (iPSC)-derived cardiomyocytes can be applied as patches to the injured myocardium [50]. The BioVAT-HF trial is currently recruiting patients with reduced ejection fraction for the implantation of engineered heart muscle [51]. The use of tissue patches for the application of stem cells to the injured heart could avoid the previously reported insufficient engraftment efficiency due to constant movement of the beating heart and a following washing-out effect [52,53,54]. On the other hand, a new strategy to enhance the regenerative activity of resident cardiac stem cell populations could be highly promising to overcome transplantation-associated issues. Interestingly, a stimulatory effect of applied exogenous stem cells to endogenous stem cells was discussed to explain the improved cardiac function after stem cell therapy [55]. The theory of a paracrine mechanism of the applied stem cells inducing remodeling processes and cardioprotection after myocardial infarct (MI) was strengthened by preclinical experiments using cell-free supernatants from cultured stem cells [56]. These observations led to a wide range of research investigating the mode of action of this stem cell-mediated paracrine signaling by examining cytokines, chemokines, exosomes and micro RNAs (miRNAs) [57]. Therefore, in order to address heart protection and repair with new stem cell-based therapeutic strategies, it is necessary to understand and target the underlying mechanisms of cardiac stem cell features such as proliferation, migration and differentiation.

Interestingly, blood plasma or serum is known as a powerful additive to enhance in vitro proliferation and survival of cellular model systems [58,59,60,61]. Moreover, the application of young blood or its derivates to old mice has resulted in enhanced tissue protection and regeneration of the aging organism [62,63,64]. However, much more research is needed to explain the relevant factors and signaling pathways underlying this effect and to develop a possible translation of this approach to the human system. When studying young blood-mediated rejuvenation of old tissues, a key interest lies in the identification of specific factors such as hormones, cytokines, chemokines or miRNAs [65]. The approaches to identify rejuvenating agents carried by young blood are highly diverse, including proteomic, metabolomic and transcriptomic methods. This review aims to summarize current knowledge about the protective effect of young blood on various organs of the mammalian body, including the heart. Next to prominent studies from the murine system, previously identified blood-borne factors and the corresponding signaling pathways will be reviewed along with the respective methods of identification. Finally, the first clinical trials investigating potential young blood-derived therapeutic perspectives will be summarized.

## 2. Parabiosis as Animal Model for Heterochronic Blood Exchange

Heterochronic parabiosis is an experimental technique where two mice of different age are surgically joined to share their circulatory systems, leading to the administration of young blood and blood borne factors to an old animal and vice versa. Aging research gained momentum in 2005, when heterochronic parabiosis experiments revealed significantly elevated regeneration of age-associated defects in old mice [66]. In detail, heterochronic parabiosis induced enhanced regeneration of muscle tissue after injury and increased proliferation of hepatocytes in old animals [66]. In 2014, the Wyss-Coray group studied age-associated alterations of hippocampal tissue and function in heterochronic parabionts and described beneficial effects of young blood on molecular, structural, and cognitive levels [64]. Further, Salpeter and colleagues detected increased proliferation of pancreatic β-cells as a rejuvenating effect of young blood in heterochronic parabiosis [67]. On the molecular level, Yousefzadeh and colleagues measured a significantly decreased transcription of senescence markers in multiple tissues of aged mice undergoing heterochronic parabiosis [68]. In 2018, Zhang and coworkers investigated correlations between iron metabolism and molecular markers of aging such as telomerase reverse transcriptase (TERT) and telomere length. By comparing heterochronic parabionts with isochronic parabionts, they detected a negative correlation of TERT in the liver, kidney and heart upon heterochronic parabiosis [69]. Despite these promising results obtained by studying heterochronic parabionts, critics repeatedly referred to the artificial characteristics of this experimental system. Next to blood-borne cytokines or chemokines, old parabionts also benefit from physiological factors such as blood pressure, which is significantly lower in young animals, and improved blood oxygenation [62,70]. Moreover, the younger organs of the young parabiont, such as lungs, liver and kidney, execute a much better removal of damaging metabolites [70]. Finally, following the 3R principles, the application of highly invasive techniques such as heterochronic parabiosis should be reduced to a minimum. Thus, new models to study the effect of young blood and its components on aged tissues are needed for an efficient translation to the human system. Here, the varying effects of young blood on different tissues of old mice underline the necessity of breaking down these complex interactions into modellable entities that can be manipulated in a controlled manner.

## 3. Identification of Blood-Borne Anti-Aging and Pro-Aging Factors

Heterochronic parabiosis experiments produced promising results, showing the high potential of young blood as treatment for age-associated malignancies. Following studies focused on the identification of specific blood-borne factors with protective or even regenerative capacities that have also been reviewed with different focuses elsewhere [65,71].

Using multi-analyte profiling, a commercially available service for quantitative, multiplexed immunoassays for 69 analytes, Chiao and colleagues detected elevated levels of matrix metalloproteinase 9 (MMP9) and monocyte chemotactic protein 1 (MCP1) in plasma samples and in left ventricle tissue of aged mice [72]. The authors thus concluded that MMP9 and MCP1 could serve as biomarkers for cardiac aging [72]. Recently, a clinical study demonstrated elevated MMP9 concentrations in serum and saliva of patients with cardiovascular disease [73]. Moreover, in MMP9 knockout mice, enhanced cardiac protection after ischemia-reperfusion-induced myocardial infarction was reported [74], while MCP1 was proposed to play a major role in the onset of cardiovascular disease [75]. Extending these conclusions, later studies introduced MCP1 and MMP9 as general biomarkers for systemic aging and not only for cardiac aging. For instance, an increased expression of MMP9 mRNA was also found in the gingiva of old (>60 years) compared to young (17–20 years) patients [76]. In addition, Yousefzadeh and coworkers utilized a Luminex platform to measure the levels of 14 circulating peptides in murine serum samples, likewise identifying MCP1 to be increased with advancing age. This group further measured elevated levels of human MCP1 in frail patients with severe aortic stenosis compared to non-frail patients [77]. In addition, comparing MCP1 plasma levels in a population of young and old non-metastatic breast cancer patients, Brouwers and colleagues detected a correlation with age but not with frailty [78]. Global analysis of RNA showed that MCP1 mRNA was strongly upregulated in glioblastoma cells when treated with tumor necrosis factor α (TNFα) in a sustained manner and at the highest level of all analyzed genes. MCP1 protein expression correlated exactly with mRNA expression [79]. Furthermore, MCP-1 induced the migration of adult neural stem cells out of neurospheres [80]. Interestingly, Pinke and coworkers detected in vitro age differences by measuring MCP1 levels in cell culture supernatants of monocytes isolated from either young or old human donors [81]. Here, ELISA assays showed increased production of MCP1 and other proinflammatory cytokines by monocytes from old (60–85 years) compared to young (20–50 years) donors. In the clinical setting, a depletion of MCP1 from the blood of prostate cancer patients was studied using the anti-MCP1 antibody Carlumab [82]. Although the treatment was well tolerated, no clinical improvements in the patients’ status could be achieved, and importantly, a permanent decrease in MCP1 serum levels could not be confirmed [83,84]. In a phase II trial, the small molecule PF-04136309, as an inhibitor of the MCP1 receptor chemokine (C-C motif) receptor 2 (CCR2), was applied to patients suffering from pancreatic ductal adenocarcinoma [85]. Unfortunately, the treatment raised concerns about pulmonary toxicity [86]. However, to the best of our knowledge, a potential protection against age-associated degeneration by depletion of MCP1 from the blood of old individuals had not been studied so far. Given the concerns raised by the clinical application of anti-MCP1 antibodies or inhibitors of the MCP1 receptor, the in vitro evaluation of new drugs should first be considered.

Next to MCP1, the chemokine Eotaxin 1, also known as C-C motif chemokine 11 (CCL11), is described to increase with age. Performing standard antibody-based multiplex immunoassays (Luminex) with plasma from heterochronic parabiotic mice, Villeda and colleagues detected a negative correlation between increasing Eotaxin 1 plasma levels and neurogenesis [63]. Notably, this group likewise measured Eotaxin 1 plasma levels in healthy human donors and described a positive correlation as well between Eotaxin 1 concentration and chronologic age [63]. However, here, a correlation coefficient of 0.4 suggested that an increase in Eotaxin 1 plasma levels alone is not sufficient to fully explain the onset of age-associated decrease in cognitive function. Further, these results from the human system were reproduced by Hoefer and colleagues, measuring via multiplex ELISA the levels of Eotaxin 1 in blood products obtained from transfusion donors. Here, fresh frozen plasma (FFP) as well as erythrocyte concentrate (EC) showed an age-dependent increase in the concentration of Eotaxin 1 [87]. A very recently published clinical trial measuring Eotaxin 1 plasma levels in healthy elderly and in people with preclinical Alzheimer’s Disease (AD) could not detect significant changes between both groups [88]. In the heart, a potential role of Eotaxin 1 in cardiovascular disease seems to be localized in the endothelial cells of coronary arteries by increasing vascular permeability and activating p38-MAPK, Stat3 and NF-kappaB pathways [89,90], but to the best of our knowledge, a direct connection between elevated Eotaxin 1 plasma levels and age-associated cardiovascular disease has not been shown so far. Interestingly, patients treated with autologous multipotent small blood stem cells (SB cells) to treat severe bone defects prior to dental implantation showed improvements of the bone defects despite increased Eotaxin 1 serum levels [91,92].

Another so-called pro-aging factor described in the literature is the small protein beta 2 microglobulin (β2M), which is present in nearly all cells and body-fluids. Increased plasma levels of β2M could be detected in aging mice via antibody-based multiplex immunoassays, while intravenous or intrahippocampal injection of β2M in young mice resulted in impaired neurogenesis and cognitive function [93]. Moreover, the study of Smith and colleagues revealed significantly increased levels of β2M in human cerebrospinal fluid (CSF) of highly aged donors [93]. Further, a slight tendency of elevated levels of β2M could also be detected in human plasma samples, although a correlation coefficient of r = 0.51 may not be sufficient to support a direct translation of these results from the murine to the human system. β2M has further been associated with frailty in elderly [94,95]. Likewise, in patients with coronary artery disease, slightly increased β2M serum levels could be detected, which also correlated with the severity of disease [96]. Similar results were obtained before in radioimmunoassays (RIAs) comparing β2M serum levels in post- and pre-menopausal women [97]. Very recently, Althubiti and colleagues described a positive correlation between age and β2M protein level, measured via ELISA assays, in human blood serum, which could also be associated with the status of oxidative stress in the respective samples. However, a direct causality between β2M serum level and oxidative stress has not been shown thus far [98]. Interestingly, single cell transcriptomic data suggest β2M is secreted by cardiomyocytes, stimulating cardiac fibroblasts after ischemia-reperfusion injury [99], possibly indicating a tissue-specific function of β2M.

Next to blood-borne factors increasing with age, other factors have been described with decreased abundance in aged individuals, thereby also potentially contributing to the onset of age-associated degeneration. One example is the growth differentiation factor 11 (GDF11), also known as bone morphogenetic protein 11 (BMP11). Loffredo and coworkers analyzed the plasma proteome of heterochronic mice by aptamer-based microarrays and described GDF11 to decline with age. Moreover, the authors achieved a significant reduction in the symptoms of age-associated cardiac hypertrophy by intraperitoneal injection of GDF11 in old mice. In iPSC-derived human cardiomyocytes, GDF11 induced increased phosphorylation of SMAD2 and SMAD3, activating the TGFβ pathway [62]. The same group later reported that administration of GDF11 also was able to reverse age-related skeletal muscle and stem cell dysfunction [100]. However, the classification of GDF11 as a potential rejuvenating factor was challenged by contradictory results presented by Egerman and colleagues. Here, the researchers performed immunoassays and detected increased levels of GDF11 dimers, the active form of GDF11, in sera of old rats and humans, which was associated with inhibited skeletal muscle regeneration in rats [101]. According to the authors, these contradictory results to the Loffredo-study may be explained with non-specificity of the used GDF11 aptamers and anti-GDF11 antibodies, which exhibited a cross reactivity with myostatin (GDF8) [101]. In a subsequent study, the Loffredo group performed additional experiments demonstrating that GDF11 as well as GDF8 declined with age in mice, rats, horses, and sheep. The authors further hypothesized that the increased signal detected by Egerman and colleagues could be caused by cross-reactivity of the GDF11 antibody with immunoglobulin, a highly abundant protein in blood that is known to increase with age [102]. Addressing this controversy, Peng and colleagues very recently developed a liquid chromatography-tandem mass spectrometry (LC-MS/MS)-based assay for the simultaneous measurement of circulating GDF8 and GDF11 in human serum samples [103]. The published results of clinical samples from healthy men between 20–90 years showed no age-associated changes in GDF8 or GDF11 [103]. Comparing individual human plasma samples derived from healthy young (< 20 years) and healthy old (>60 years) donors via commercial ELISA assays, we detected only a slight decrease in the plasma protein levels of GDF11 as well as Eotaxin 1, while MCP 1 remained unchanged [25]. However, in patients with ischemic heart disease, higher plasma levels of GDF11 have been associated with lower risk of cardiovascular events and death [104], indicating that GDF11 is more likely associated with cardiovascular risk than with aging.

A further factor which has been shown to be associated with age is the hormone oxytocin. Although the function of oxytocin and its receptor in lactation, parturition and social behavior is well studied, its anti-aging effects in terms of regeneration or protection against degenerative agents remained unclear. Elabd and coworkers measured declining oxytocin plasma levels in aging mice via enzyme immunoassays (EIA) together with decreased protein expression of oxytocin receptor in satellite cells, but not in skeletal muscle cells [105]. This decrease in oxytocin expression was accompanied by inhibited skeletal muscle regeneration, which could be reversed by systemic delivery of oxytocin [105]. Moreover, oxytocin has been shown to induce the proliferation of activated mouse satellite cells via MAPK/ERK signaling in vitro [105]. Interestingly, oxytocin has further been shown to exert a protective effect with antiapoptotic and anti-inflammatory properties on cardiomyocytes [106]. Further, in the developing rat heart, increased expression of oxytocin and its receptor has been reported, but decreases postnatally to lower levels [107]. Moreover, oxytocin was also successfully applied in myocardial differentiation of adult murine progenitor cells [20]. In the human system, declining plasma levels of oxytocin in osteoporotic women compared to non-osteoporotic women were reported by Elabd and colleagues applying enzyme immunoassays [108]. Likewise, a cross-sectional study measuring oxytocin serum levels in 1097 postmenopausal women showed high oxytocin serum levels to be associated with high bone mineral density [109]. However, more research is needed to clearly confirm age-dependent plasma levels of oxytocin and to describe its protective effects on different stem cell populations particularly in the human system. Interestingly, a randomized controlled trial investigating the effects of intranasal application of oxytocin on psychological well-being of residentially housed older adults revealed no effects on the cardiovascular state as a side measurement [110].

Another hormone declining with age in humans and mice is apelin. Apelin is produced by myofibers, and its production is stimulated by exercise-associated muscle contraction, but decreasing apelin synthesis in the skeletal muscle as well as decreasing levels in plasma could be measured during aging in mice and humans [111]. Further, a reduced expression of the apelin receptor was detected in muscle stem cells of old mice [111]. Likewise, apelin supplementation resulted in restored skeletal muscle function with enhanced biogenesis of mitochondria [111]. Apelin infusion further resulted in a reduction of age-associated cardiac hypertrophy in mice and a reduction of senescence markers [112]. In the clinical setting, interesting findings were achieved by the application of autologous bone marrow mononuclear cells (BMMCs) to patients with severe heart failure secondary to myocardial infarction. Here, generally lower serum levels of apelin were measured in all patients compared to healthy subjects. After transplantation of BMMCs, a significant increase in apelin accompanied by improvements in cardiac function was detected compared to patients receiving standard medical treatments [113]. The researchers thus speculated that apelin may act as a paracrine factor enhancing cardiac repair.

The Wyss-Coray group administered human umbilical cord blood plasma to aging mice, detecting enhanced synaptic plasticity that led to improved cognitive function. Further protein microarray-based comparisons of human umbilical cord plasma with plasma of young and old adult donors showed significantly enriched amounts of the tissue inhibitor of metalloproteinase 2 (TIMP2) in umbilical cord plasma. Moreover, depletion of TIMP2 from cord blood plasma in following experiments revealed a connection between TIMP2 abundance and the neuroprotective effects of cord blood plasma [114]. The Castellano study focused on hippocampal aging, but TIMP2 deficiency has also been shown to inhibit cardiac remodeling processes after myocardial infarction in mice via inhibition of membrane type 1 matrix metalloproteinase (MT1-MMP) [115]. TIMP2 has further been proposed to be involved in homing mechanisms of mesenchymal stem cells [116]. In contrast, elevated plasma levels of TIMP1, TIMP2 and TIMP4 have been associated with higher risk for major adverse cardiac events after acute myocardial injury in a clinical cohort of 1313 patients [117]. More research is necessary to clarify the role of TIMP2 in cardiac and neural protection.

Another circulating factor that has been shown to decline with age in humans, rhesus monkeys and mice is the bone-derived hormone osteocalcin (OCN) [118,119,120]. Mera and coworkers detected significantly decreasing OCN plasma levels with increasing age in male and female mice via ELISA assays. Interestingly, a study by Khrimian and colleagues linked the decrease in OCN plasma levels to a decrease in cognitive functions and showed that OCN depletion in plasma samples from young mice partially reverses the beneficial effects on cognitive function when applied to old mice, while systemic administration of OCN improved the cognitive abilities of old mice [118]. In the human system, declining OCN serum levels were linked to left ventricular systolic dysfunction in men but not in women, might suggesting a sex-specific effect of OCN [121].

**Figure 2:**
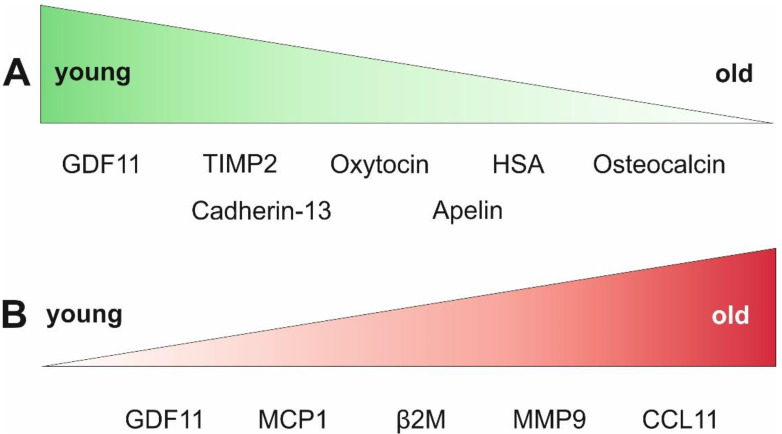
Overview of the currently known blood-derived factors with age-dependent serum levels and impact on various age-associated malignancies. (**A**) Factors enriched in blood samples from young individuals but declining with age. (**B**) Factors with low abundancy in young individuals but with increasing levels with age.

Using tandem mass spectrometry, Yang and colleagues detected declining plasma levels of cadherin-13 in aged mice, while intraperitoneal injection of cadherin-13 delayed age-associated bone loss [122]. This effect was explained by the capacity of cadherin-13 to inhibit the differentiation of bone marrow-derived macrophages to osteoclasts [122]. Figure 2 provides a graphical overview about the rejuvenating or pro-aging factors discussed herein.

Although most research to identify potential blood-borne factors influencing aging is focused on proteomic approaches, the lipidome and the metabolome were also considered to be involved. However, to the best of our knowledge, a contribution of either the lipidome or the metabolome to the onset of age-associated degeneration has not been shown thus far. As one example, Loffredo and coworkers performed metabolomic and lipidomic profiling of plasma samples from heterochronic parabionts using LC-MS/MS analysis. However, this method did not reveal significant differences between heterochronic compared to isochronic parabiotic mice [62]. In addition, Yousef and colleagues removed the metabolites from blood plasma from aged mice by dialysis before administration to young mice while still observing reduced neural progenitor activity in the hippocampi. The researchers thus suggested that proteins, and not metabolites or small molecules, would be the active circulatory signal inducing age-related degenerations in the brain [123].

Despite the promising identification of diverse potentially protective or even rejuvenating components in young blood plasma (Table 1), most of the effects seen in the animal system have not been tested in the human system so far. To overcome this transitional gap and to understand the effects of young human blood components at a cellular and molecular level, suitable model systems are needed. Using iPSC-derived specialized cell types, multicellular organotypic models can be established, such as organ-on-a-chip systems, which are increasingly acknowledged in drug testing with a special focus on cardiotoxicity [124]. However, a main limitation of iPSC-derived model systems is the low grade of maturity compared to primary adult heart tissue [124,125]. In contrast, primary adult human stem cells are crucial actors in tissue homeostasis and regeneration and should be considered as promising cellular model systems for the investigation of blood plasma-mediated effects in the human system.

In vitro experiments performed by Walenda and coworkers showed that serum taken from patients after high dose chemotherapy significantly increased the proliferation of CD34^+^ hematopoietic stem and progenitor cells (HSPCs). Interestingly, using LC/MS and GC/MS, the researchers could detect changes in the metabolomic profile of serum samples before and after chemotherapy, but these metabolites seemed not to be the active components inducing HSPC proliferation [126]. However, via microarray analysis, this model revealed changes in the abundance of 23 miRNAs in serum samples after chemotherapy, which led to the identification of miRNA-320c as a potentially active factor in the induction of serum-mediated HSPC proliferation [126]. Along with miRNAs as potential mediators of serum-induced effects, exosomes are discussed as carriers for anti-aging or pro-aging factors in the blood. In recent years, numerous studies have investigated the potential of exosomes derived from young plasma or from stem cell culture supernatants in regenerative medicine [127,128]. In murine model systems of myocardial infarct, the intramyocardial or intravenous injection of stem cell-derived exosomes resulted in significantly improved cardiac repair [129,130]. Furthermore, in vitro assays were developed to investigate the underlying mechanisms of the beneficial effects of exosomes on tissue repair. For instance, Xu and colleagues applied mesenchymal stem cell-derived exosomes to cultured neonatal rat cardiomyocytes and reported significantly reduced apoptosis after hypoxic injury [131]. Further, Bobis-Wozowicz and coworkers showed that iPSC-derived exosomes are able to induce proliferation and enhance the cardiac and endothelial differentiation potential of human heart-derived mesenchymal stromal cells [132]. Lee and colleagues recently demonstrated that exosomes isolated from serum samples of young mice significantly improved pathological markers of Huntington’s disease (HD) in an in vitro cellular model of HD [133].

As the most abundant protein in blood serum, human serum albumin (HSA) exerts multiple functions in the colloidal osmotic pressure of blood and binding and transport of ions, toxins, or cytokines [134,135]. In addition, serum albumin has been shown to have antioxidative properties [136]. Interestingly, a slight decrease in the level of serum HSA during aging was suggested [137,138]. In the cardiovascular system, a meta-analysis by Wang and colleagues revealed an association between low levels of HSA with an increased risk of atrial fibrillation [139]. In the nervous system, Costa and Páez comprehensively summed up current knowledge about the use of plasma exchange with albumin replacement as a treatment for AD patients [140]. Likewise, we very recently described significant protection against oxidative stress in mouse hippocampal slice cultures as well as in adult human stem cell-derived human neurons [141] after application of HSA.

These data demonstrate the great potential of in vitro models using human stem cells to test the effects of blood plasma or its single components. Moreover, omics approaches are widely used to collect data helping to identify active factors. Likewise, in previous studies, we examined proliferation, senescence and migration of human blood serum-treated adult human cardiac stem cells (hCSCs) [25,142]. Although we could detect significantly increased proliferation and migration next to significantly decreased senescence in comparison to untreated cells, a direct conclusion about the underlying signaling pathways could not be drawn using these assays. Here, the bioinformatic analysis of the global transcriptome of serum-treated hCSCs revealed potential molecular pathways to be affected by serum treatment. Subsequent inhibitor assays revealed p38-MAPK to be involved in the cellular response of hCSCs to human serum [25,142]. Further, using a microfluidic cultivation chamber, we performed migration analysis of human cardiac stem cells on the single cell level, revealing a signaling cascade via p38-MAPK and phosphorylated heat shock protein 27 (phosphoHSP27) in response to serum treatment [142].

The use of single cell analysis combined with omics technologies to understand the regenerative response of human (stem) cell populations further seems promising regarding the highly interconnected pathways affected by systemic interventions. Very recently, a consortium around the Wyss-Coray group investigated the cell type-specific effects of heterochronic parabiosis on gene expression at the single cell level [143]. Here, especially adipose-derived mesenchymal stem cells and hematopoietic stem cells, as well as hepatocytes seemed to be most responsive upon treatment with young or old blood, respectively. However, when interpreting these results, the age-independent effect on the surgical procedure of parabiosis itself must be considered. Interestingly, an overall reduction in gene expression was reported for all cell types upon aging. In our own in vitro studies, we likewise detected an overall reduction in differential gene expression of human cardiac stem cells treated with blood serum of old human donors (age > 60 years) [25]. The highly complex results of the Pálovics study once more underline the necessity to investigate organ- and cell type-specific effects of young blood and its derivates on the aging human body. Especially in research regarding cardiac regeneration, the use of single cell analysis seems to be extremely promising to evaluate the responses of the different cardiac cell types to the stimulus of young blood serum.

**Table 1:** Summary of previously identified blood-borne anti-aging or pro-aging factors. ↓: factor is decreased with age. ↑: factor is increased with age.

Factor/Pathway	Effect	References
CCL11 (Eotaxin 1)↑	Elevated plasma levels are associated with decline in neurogenesis and impaired learning and memory; increases vascular permeability and activates p38-MAPK, Stat3 and NF-kappaB pathways in human coronary artery endothelial cells; no significant differences in Eotaxin 1 plasma levels in healthy elderly and in people with preclinical AD	[63,88,89,90]
β2M↑	Induces impaired neurogenesis and cognitive function in mice; stimulation of cardiac fibroblasts after ischemia-reperfusion injury; elevated plasma levels are associated with frailty	[63,93,94,95,99]
GDF11↓↑	Contradictory results: reduction in the symptoms of age-associated cardiac hypertrophy in old mice, increase in SMAD2- and SMAD3 phosphorylation in cardiomyocytes; reversal of age-related skeletal muscle and stem cell dysfunction; inhibition of skeletal muscle regeneration in rats	[62,100,101,102,104]
Oxytocin↓	Application of oxytocin leads to activated skeletal muscle regeneration, proliferation of mouse satellite cells via MAPK/ERK, antiapoptotic and anti-inflammatory effects on cardiomyocytes; supports myocardial differentiation of adult murine cardiac progenitor cells; high oxytocin serum levels were associated with high bone mineral density in postmenopausal women	[20,105,106,109]
Apelin↓	Decreasing levels in plasma of aging mice and humans accompanied with reduced expression of the apelin receptor in mice; apelin supplementation resulted in restored skeletal muscle function with enhanced biogenesis of mitochondria and reduction of age-associated cardiac hypertrophy in mice; generally lower serum levels of apelin were measured in patients with severe heart failure secondary to MI compared to healthy subjects	[111,112,113]
Cadherin-13↓	Declining plasma levels in aged mice, while intraperitoneal injection of cadherin-13 delays age-associated bone loss; inhibition of the differentiation of bone marrow-derived macrophages to osteoclasts	[122]
TIMP2↓	Application of TIMP2 leads to enhanced synaptic plasticity and improved cognitive function in mice; TIMP2 deficiency inhibits cardiac remodeling processes after myocardial infarction in mice via inhibition of membrane type 1 matrix metalloproteinase; involved in homing mechanisms of human mesenchymal stem cells; elevated plasma levels of TIMP1, TIMP2 and TIMP4 have been associated with higher risk for major adverse cardiac events after acute myocardial injury in humans	[114,115,116,117]
Osteocalcin↓	Decrease in OCN plasma levels is linked to a decrease in cognitive functions; systemic administration of OCN improves the cognitive abilities of old mice; declining OCN serum levels were linked to left ventricular systolic dysfunction in men	[118,119,121]
MMP9 ↑	Biomarker for cardiac aging; knockout leads to enhanced cardiac protection after myocardial infarction in mice; elevated MMP9 concentrations were measured in serum and saliva of patients with cardiovascular disease; increased expression of MMP9 mRNA in the gingiva of old (>60 years) compared to young (17–20 years) patients	[72,73,74,76]
MCP1↑	Biomarker for cardiac aging; proposed to play a major role in the onset of cardiovascular disease; depletion of MCP1 from the blood using the anti-MCP1 antibody Carlumab did not result in a permanent decrease in MCP1 serum levels	[72,75,77,82,83,84]
Serum albumin↓	Low serum albumin levels are associated with decreased antioxidative properties; application of serum albumin leads to protection against oxidative stress in mouse hippocampal slice cultures and in human neurons; low serum albumin levels are associated with increased risk of atrial fibrillation	[136,139,141]
Exosomes	Mesenchymal stem cell-derived exosomes reduce apoptosis after hypoxic injury in neonatal rat cardiomyocytes; iPSC-derived exosomes induce proliferation and enhance the cardiac and endothelial differentiation potential of human heart-derived mesenchymal stromal cells; exosomes from serum of young mice significantly improved pathological markers of Huntington’s disease	[131,132,133]
P38-MAPK pathway	Increased proliferation and migration of hCSCs	[25,142]

## 4. Clinical Trials Assessing the Effects of Young Blood

Although the idea of young blood as a source of diverse beneficial factors that ameliorate the phenotypic manifestations of aging is tempting, a recent review by Hofmann reminded us that a translation of these promising results from the murine to the human system has so far not been successful (Hofmann 2018). Facing this challenge, the first clinical trials were conducted investigating the effects of blood or plasma from young donors administered to old participants. Interestingly, the majority of these trials focused either on general safety and feasibility or on the improvement of the symptoms of neurodegenerative diseases. To the best of our knowledge, clinical studies assessing a protective effect of young blood on the heart and cardiovascular system have not been published so far (Table 2).

Edgren and colleagues performed a retrospective cohort study using data from the Scandinavian Donations and Transfusions database assessing a potential association between donor age and sex and the recipient survival rates after red blood cell transfusion. Here, data of 968,264 patients did not show a connection between donor age and sex and the survival rates of the recipients [144].

The Wyss-Coray group performed a phase I clinical study testing safety, tolerability, and feasibility of FFP from young donors (age 18–30) infused into 18 patients with AD [145]. This study aimed to translate the promising results in mice to the human system and showed the general safety and feasibility of the procedure. Moreover, a clinical study with the blood plasma-derived product GRF6019, a plasma fraction of about 400 proteins, is already finished, but peer-reviewed results are only available regarding safety and tolerability [146], while detailed readout of the respective manifestations of AD within these patients has not yet been published.

A comparable study applying young blood plasma of male donors between 18–25 years old to Parkinson’s disease (PD) patients was likewise conducted. Here, next to safety measurements, laboratory makers of Parkinson’s disease as well as the progression of cognitive, mood and motor impairments were monitored. Interestingly, secondary outcome measures revealed slight but significant improvement in phonemic fluency, indicating that young blood plasma could be a powerful additive to conventional therapies. Further, blood levels of the inflammatory marker TNFα were elevated before treatment with young blood plasma but decreased after 4 weeks post transfusion. However, young blood plasma seemed to have no effect on the levels of other inflammatory markers such as interleukin 6 (IL6) [147]. Here, more independent data would be useful to evaluate the impact of young human blood plasma on the inflammatory marker profile of elderly or diseased individuals.

Several clinical trials were announced regarding the measurement of age-associated biological and physiological scores such as markers of frailty or biomarkers of inflammation, oxidative stress or adverse hormone status. For instance, in 2017, a clinical study was planned to investigate the effects of umbilical cord blood on recipients between 50–80 years of age. Here, extensive measurements were planned to assess the level of frailty via inflammatory biomarkers, adrenal cortical hormones, oxidative stress status, telomere length and the extent of DNA damage [148]. However, the actual recruitment status is unclear, and potential results have not been published yet. A similar trial was planned in 2015 to evaluate the clinical effects of fresh cord blood, frozen cord blood and frozen plasma on recipients with a diagnosis of pre-frailty [149]. Likewise, the recruitment status of this study is unknown. Another clinical trial to assess the safety and efficacy of human umbilical cord blood plasma for age-related cognitive decline was announced in 2020. Here, next to the safety of umbilical cord blood plasma for recipients between 65–85 years, secondary outcome measures should investigate executive function and working memory. However, the results are not accessible since the study is not yet recruiting [150]. Further, a clinical trial that started very recently plans to address the use of plasmapheresis for treatment of age-related frailty. Here, therapeutic plasma exchange is planned to be applied during a plasmapheresis procedure. In addition, serum albumin is to be added to replenish albumin levels in the recipients. In this study, patients between 50–95 years of age with clinical signs of frailty will be included. As an outcome measure, the overall fitness and frailty of the recipients is planned to be measured according to the CSHA Clinical Frailty Scale. The study is planned to be completed in 2025, and the results may help to extend the current knowledge about the role of serum albumin levels in age-associated degenerations [151]. Interestingly, we recently performed experiments investigating neuroprotective effects of human blood serum and serum albumin in oxidatively stressed murine hippocampal slice cultures. We could detect comparable effects of human serum and serum albumin alone, indicating a proper serum albumin level to be crucial for the observed neuroprotective action of human blood serum [141]. Another study was planned to investigate potential beneficial effects of young blood plasma infusions from donors aged between 18–30 years to patients with acute stroke [152]. However, also for this study, the recruitment status is unknown and results are not available. The company Ambrosia announced a clinical trial with 200 participants receiving infusions of young blood plasma from donors aged between 16–25 years [153]. The aim of this study was to measure a panel of diverse biomarkers such as blood cell counts, blood chemistry, the levels of immunoglobulins and a wide range of chemokines and cytokines including β2M, Eotaxin 1, MCP1 and MMP9. While data on the clinical outcome would be highly interesting to gain more insights on how young blood plasma affects old individuals, again, the results of this trial have not been posted. Of note, in 2019, the company temporary halted operations after the Food and Drug Administration (FDA) released a statement expressing concerns about the lack of clinical evidence for benefits and associated safety risks related to infusions with young blood plasma for profit [154]. In summary, although encouraging results occasionally could be reported in the clinical setting, more data from clinical trials are needed for a profound answer to the question of whether a blood plasma-mediated regeneration of the aged organism can be observed even in the human system.

Overall, most clinical research investigating the effects of young blood on the aging system and age-related pathologies has been focused on neurodegenerative diseases. Bearing in mind the previously mentioned preclinical research showing cardioprotective effects of young blood and its components, a clinical investigation of these effects would be highly interesting. In 2020, a trial was planned to study the safety and efficacy of allogeneic young plasma infusion in geriatric patients with heart failure, but this trial was withdrawn before its start [155]. To the best of our knowledge, more clinical trials investigating the effects of young blood plasma or its derivates on cardiac repair have not been announced thus far.

**Table 2:** Summary of clinical trials.

Product	Target/Measurements	Outcome	Reference/ ClinicalTrials.gov Identifier
Young plasma (male, 18–30 years)	Safety of intravenously administered young plasma for patients with AD	Safety and feasibility of infusions with young plasma for people with AD have been demonstrated	[145,156] NCT02256306
GRF6019, a plasma-derived product	Safety, tolerability, and feasibility of intravenous infusion in patients with mild to moderate AD	GRF6019 is safe and well tolerated; patients experienced no cognitive decline	[146,157] NCT03520998
Young plasma (male, 18–25 years)	Safety of young plasma for patients with Parkinson’s disease; laboratory makers of PD; progression of cognitive, mood and motor impairments	Primary outcome: safety and feasibility of infusions with young plasma for people with PD. Secondary outcome: slight but significant improvement in phonemic fluency; decreased blood levels of TNFα	[147,158] NCT02968433
Umbilical cord blood plasma	Safety for intravascular administration for patients between 50–80 years; physiological markers of frailty or other age-related biological measures	Recruitment status unknown; No outcome published	[148] NCT03229785
Umbilical cord blood plasma	Umbilical cord blood plasma infusion (50 mL) in elderly adults (65–85 years) with age-related cognitive decline Assessment of: safety, executive function, working memory	Not yet recruiting; No results posted	[150] NCT04566757
Fresh umbilical cord blood, frozen umbilical cord blood, frozen plasma	Fresh cord blood, frozen cord blood and frozen plasma intravenously administered to recipients (>55 years) with a diagnosis of pre-frailty Assessment of: safety, cardiac output, biomarkers for oxidative stress, inflammation and immune response, methylation, mitochondrial DNA copy number, growth factors, antioxidant capacity, hormone status, DNA damage, metabolite	Recruitment status unknown; No results posted	[149] NCT02418013
Plasmapheresis	Safety and feasibility of plasmapheresis/therapeutic plasma exchange with albumin in patients with age-related frailty (50–95 years)	Enrolling by invitation; No results posted (estimated primary completion date: April 2025)	[151] NCT05054894
Young blood plasma (male, 18–30 years) Old blood plasma (male, 40–55 years)	Efficacy and safety of young plasma for patients with acute stroke	Recruitment status unknown; No results posted	[152] NCT02913183
Young blood plasma (16–25 years)	Infusion of young plasma in healthy recipients older than 30 years. Measurement of clinical biomarkers of aging.	No results posted	[153] NCT02803554
Plasma from young donors	Safety and feasibility of plasma infusions in geriatric patients (65–80 years) with heart failure with preserved ejection fraction (HFpEF)	Withdrawn before start	[155] NCT04241159

## 5. Conclusions

The use of young blood or young blood-derived factors was sufficient to rescue age-associated degenerations in diverse organs, including the heart, in the murine body. Further research regarding the identification of its active factors and the underlying molecular mechanisms may promote the design of new drugs for tissue-specific regeneration. Although it would be tempting to slow down or even reverse the progression of age-associated diseases in the human system, scientific evidence from clinical trials is very limited at the moment. Especially with regards to cardiac repair, clinical studies are not available. The lack of information about potential clinical benefits induced by the administration of young plasma or its derivates partially represents the lack of knowledge about the underlying mechanisms. To close this gap, more research is necessary to understand the underlying effects of young blood and the affected molecular pathways in a cell type-specific manner. In this case, in vitro cultures of adult human stem cell populations are a useful and easy-to-manipulate setup to obtain cell type-specific data from the human system on a molecular level. Moreover, not only individual factors but rather the interaction or the ratio of different blood-derived factors should be investigated in vitro to elucidate the mode of action on special stem and progenitor cell populations.

## Data Availability

Not applicable.
